# Estimating the Population Size and Genetic Diversity of Amur Tigers in Northeast China

**DOI:** 10.1371/journal.pone.0154254

**Published:** 2016-04-21

**Authors:** Hailong Dou, Haitao Yang, Limin Feng, Pu Mou, Tianming Wang, Jianping Ge

**Affiliations:** Ministry of Education Key Laboratory for Biodiversity Science and Engineering and College of Life Sciences, Beijing Normal University, Beijing, China; University of Illinois at Urbana-Champaign, UNITED STATES

## Abstract

Over the past century, the endangered Amur tiger (*Panthera tigris altaica*) has experienced a severe contraction in demography and geographic range because of habitat loss, poaching, and prey depletion. In its historical home in Northeast China, there appears to be a single tiger population that includes tigers in Southwest Primorye and Northeast China; however, the current demographic status of this population is uncertain. Information on the abundance, distribution and genetic diversity of this population for assessing the efficacy of conservation interventions are scarce. We used noninvasive genetic detection data from scats, capture-recapture models and an accumulation curve method to estimate the abundance of Amur tigers in Northeast China. We identified 11 individual tigers (6 females and 5 males) using 10 microsatellite loci in three nature reserves between April 2013 and May 2015. These tigers are confined primarily to a Hunchun Nature Reserve along the border with Russia, with an estimated population abundance of 9–11 tigers during the winter of 2014–2015. They showed a low level of genetic diversity. The mean number of alleles per locus was 2.60 and expected and observed heterozygosity were 0.42 and 0.49, respectively. We also documented long-distance dispersal (~270 km) of a male Amur tiger to Huangnihe Nature Reserve from the border, suggesting that the expansion of neighboring Russian populations may eventually help sustain Chinese populations. However, the small and isolated population recorded by this study demonstrate that there is an urgent need for more intensive regional management to create a tiger-permeable landscape and increased genetic connectivity with other populations.

## Introduction

The Amur tiger (*Panthera tigris altaica*), a flagship species and a top predator, plays a vital role in maintaining the stability and health of the coniferous and broadleaved mixed forests of Northeast Asia [1, 2]. The Amur tiger once widely inhabited Northeast China, the Far East of Russia and the Korean Peninsula before the 20^th^ century [3–5]. Habitat loss and intensive hunting greatly reduced the tiger population and fragmented its distribution into small patches. As a result, only approximately 500 individuals remain in the wild and more than 95% of them dwell in the Sikhote-Alin Mountains of the Russian Far East [6], with an effective population size less than 30 [7, 8]. Currently, Amur tigers in China are found in several small and isolated patches scattered in Jilin and Heilongjiang provinces near Russia [9–12]. These tigers have had to compete with the local people to survive because human activities such as agriculture, livestock grazing and forest logging have seriously affected their presence and abundance [5, 10, 13]. Among these scattered populations, the Hunchun tiger population is the largest one in eastern Jilin province and is believed to be a part of an adjacent tiger population in southwest Primorye Krai of Russia. Hunchun Nature Reserve and adjacent forest is considered a promising area for tiger restoration in Northeast China if inviolate land is maintained [3, 10, 14]. However, the current demographic status of these populations is uncertain. Information on the abundance, distribution and genetic diversity of this population for assessing the efficacy of conservation interventions is scarce.

Reliable estimates of population size are critical in the recovery of small populations of threatened species as these estimates provide the basis for predicting the persistence of these populations and their ability to adapting to environmental changes [15, 16]. Using demographic data, policy makers and conservationists can formulate corresponding management actions, especially for endangered species. Previous tiger demographic studies in China have mostly used traditional field survey methods, such as the line transect method, footprint or scratch counting, and snow tracking, that does not accurately identify individuals and predict the spatial distribution and structure of the population [12, 17]. Although a recent study based on camera-trapping techniques could precisely estimate population size [13, 18], detailed information on the genetic status of this population could not be obtained.

Noninvasive genetic sampling has been considered an effective supplementary method for monitor populations, especially for elusive and rare species so that genetic diversity can be assessed and population persistence can be estimated [19–21]. Behavioral features (i.e., basic home range sizes) might also be estimated. With the development of microsatellite loci in cats [1, 22], this DNA-based technique has been widely used in tiger surveys to provide scientific and diagnostic analyses for the conservation and management of tigers in different regions [19, 23, 24]. In Russia, 12 individual tigers were identified in the southwest Primorye Krai using noninvasive genetic samples [23]. In China, a recent study identified five tiger individuals by analyzing nine scats collected in Hunchun Nature Reserve [25]. However, these previous results have documented only the presence of Amur tigers; these studies have not been able to provide a valid estimation of population size because of limited sample size and a limited sampling area.

In this study, we conducted a thorough survey to collect tiger scats in three nature reserves of Northeast China. Our aims were to identify individuals, estimate population abundance and assess genetic diversity using non-invasively obtained genetic data. We assessed whether tiger abundance was comparable to the abundance recorded from camera-trapping surveys conducted under a formal capture-recapture sampling framework [18]. Additionally, we reconstructed the putative home ranges of some identified tigers on the Chinese side based on the spatial distribution of genotyped scats. Finally, we examined the general conservation implications of our findings for monitoring wild Amur tiger populations and understanding their population status in China.

## Material and Methods

### Ethics Statement

China’s State Forestry Administration approved this study as a part of the long-term Tiger Leopard Observation Network (TLON). Jilin Province Forestry Bureau and the Forestry Industry Bureau of Heilongjiang Province approved permits for the work conducted. Beijing Normal University conducted this study in collaboration with the administrations of the local protected areas. Scat-collection surveys used non-invasive technology and did not involve direct contact with animals.

### Study Area

**Fig 1:**
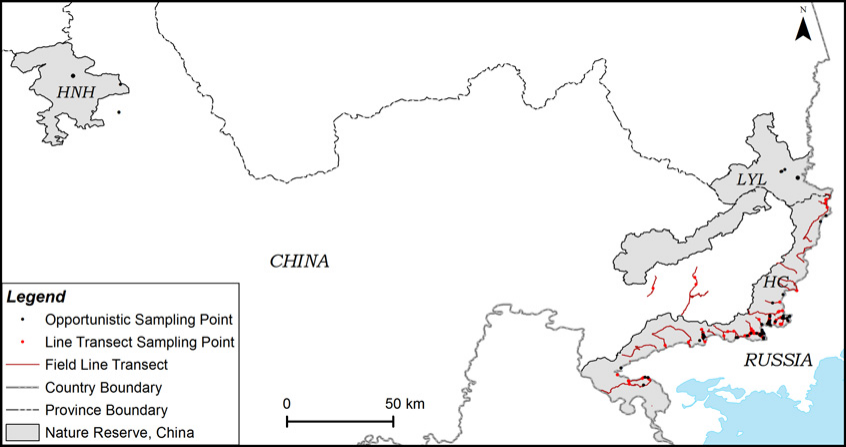
Locations of tiger fecal samples collected in Huangnihe (HNH), Hunchun (HC) and Laoyeling (LYL) Nature Reserve. Black points indicate the positions of scats that were opportunistically collected by field staff between April 2013 and May 2015. Red points indicate the positions of scats that were collected using the line transect sampling method in HC between December 2014 and February 2015.

The scats were collected from three major tiger nature reserves in east Jilin and Heilongjiang, Northeast China: Hunchun reserve (HC), Laoyeling reserve (LYL) and Huangnihe reserve (HNH) (Fig 1). HC and LYL are next to each other and border Russia’s Land of Leopard National Park, forming a trans-boundary conservation landscape. HNH is located in the interior of Jilin Province, approximately 270 km west of HC and LYL. These reserves are on a rugged, mountainous landscape with elevations ranging from 5 to 1477 m. The whole region has a typical temperate and monsoonal climate with mean annual precipitation of approximately 580–618 mm. The annual average temperature ranges from 3.90–5.65°C, and the frost-free period lasts for approximately 110–160 days/year depending on latitude, longitude and topography. Vegetation is classified as mixed Korean pine (*Pinus koraiensis*)—deciduous forest dominated by Korean pine, Manchurian ash (*Fraxinus mandshurica*), Manchurian walnut (*Juglans mandshurica*), Mongolian oak (*Quercus mongolica*), and several species of maple (*Acer* spp.) and birches (*Betula* spp.). Because of long-term repeated disturbances (i.e., logging, fires), the original vegetation has been almost entirely replaced by secondary forests dominated by Mongolian oaks, shrub land and spruce-fir coniferous forests at higher elevations [17]. In addition to the Amur tiger, a diverse population of mammals inhabit this reserve including large carnivores such as the Amur leopard (*Panthera pardus orientalis*), black bear (*Ursus thibetanus*) and brown bear (*Ursus arctos*), and prey species such as sika deer (*Cervus nippon*), Siberian roe deer (*Capreolus pygargus*), and wild boar (*Sus scrofa*) [13]. The forest landscape is fragmented with numerous villages, farmlands and ranches leading to a conflict between tigers and local residents [26].

### Scat Collection

Two scat collection approaches were used in this study. The first approach was a routine opportunistic approach where fresh scats were collected along logging roads, dirt roads and forest trails by field staff when they periodically monitored Amur tigers and Amur leopards in the study area from April 2013 to May 2015. The second approach was more systematic: specifically, we collected scats along a predefined transects during the winter of 2014–2015 in HC. To increase sampling efficiency, we established 18 transects that the tigers regularly used based on our long-term camera-trapping data. Each transect was inspected by a team of two trained members at six-day intervals. The total length of transects was 162 km, covering an area of approximately 1000 km^2^. Two-member team collected scats were conducted along the line transects for 54 consecutive days between December 2014 and February 2015. The size of scats and associated tracks and scrapes were measured. Each scat was placed in a ziploc bag by hand; disposable gloves were worn at all times while handling scat. The samples were stored in a refrigerator at -20°C until further analysis.

### Genetic Methods

DNA was extracted from the scats using the QIAamp Fast DNA Stool Mini Kit (QIAGEN, Inc.) as described in Russello et al. [27]. Negative controls were included in every fecal DNA extraction under sterilized conditions to avoid cross-contamination of samples. Due to the difficulties in differentiating tiger scat from those of sympatric leopard by size, shape and other associated signs, we first identified the sources of these scats following the methods of Sugimoto et al. [28] by amplifying a 156-bp mitochondrial DNA Cytochrome *b* gene (*Cytb*) fragment for leopards and a 271-bp fragment for tigers before genotyping. A distinct target band was detected by agarose gel electrophoresis at least twice after being screened three times to identify the species. The tiger-positive samples were genotyped at thirteen nuclear microsatellite loci (FCA304, F42, FCA77; FCA105, FCA441; FCA161, D06, FCA723; 6HDZ089, FCA32, FCA94; F124, FCA391), which were selected for their highly polymorphic information content, ease of amplification and low error rate determined in previous tiger studies [19, 23, 29]. Three of the microsatellites were omitted because they were not polymorphic (FCA32 and FCA391) or there was a low rate of amplification (FCA723). Ten loci were combined and amplified in five multiplex reactions to increase efficiency and reduce costs.

Polymerase Chain Reaction (PCR) for individual identification was performed at a 15-μl volume containing 7.5 μl KHBE PCR MasterMix (Beijing KHBE, Inc.), 0.2 μm fluorescently labeled forward primer (labeled 5’ FAM, HEX, TAMRA and ROX), 0.2 μm usual reverse primer, 1 μg bovine serum albumin (TaKaRa) and 2 μl DNA template. Amplification was performed in a Applied Biosystems Veriti Thermal Cycler with an initial denaturation at 94°C for 5 min, followed by 45 cycles of 94°C for 1 min, 52–59°C for 30 sec, 72°C for 30 sec, and a final extension at 72°C for 10 min. PCR products were separated using capillary electrophoresis on an ABI 3730XL automatic sequencer (Applied Biosystems, Inc.). Allele data were obtained using GeneScan-500 LIZ Size Standard and scored on GeneMapper software (version 4.1). Because of the poor quantity of fecal DNA and high genotyping errors, we adopted the multi-tube approach to improve detection accuracy [30]. Each PCR reaction was repeated three times at first. If the sample showed a consensus genotype without ambiguous amplifications, allelic dropout (ADO) and false alleles (FA), additional reactions were not conducted. Otherwise, two to four additional amplifications were conducted until both alleles of a heterozygous genotype were detected at least three times or one allele of a homozygous genotype was detected at least five times. The sex of the identified individual was determined by amplifying the introns of the zinc finger on the X chromosome and the DEAD box polypeptide on the Y chromosome [28]. Every sample was also amplified three times to ensure the accuracy of sex identification. We used the threshold method in the software GLMELET [31] to calculate error rates of ADO and FA and to construct consensus genotypes for individual identification.

### Data Analysis

Mstools (available at http://ms.biomed.cas.cz/MSTools/) was used to compare and identify matching genotypes to consensus genotypes. Fecal samples that matched at a minimum of nine loci were pooled to create consensus genotypes. Probability of identity (PID) was used to test whether the number of loci were adequate for distinguishing unrelated individuals, and siblings were estimated using a corrected equation for small populations in the program GIMLET [31]. The smaller the value of the PID, the higher the sensitivity of molecular markers for genetic studies.

Genetic diversity for ten microsatellite loci was measured as the mean number of alleles per loci (N_A_), expected heterozygosity (H_E_), observed heterozygosity (H_O_) and polymorphism information content (PIC) using FSTAT2.9.3.2 [32]. Hardy-Weinberg equilibrium (HWE) and linkage disequilibrium (LD) were tested using sequential Bonferroni corrections in the program GENPOP (version 3.4) [33, 34].

We used individual genetic capture-recapture data from scats collected by the transect method during the winter of 2014–2015 in HC to estimate population size, as has been successfully used in a number of recent estimates of the abundance of felid species [23, 35]. In the 54-day window, the standard X matrix of individual capture histories was generated by collecting data every six days to increase detection probabilities and improve estimates, as suggested by previous studies examining elusive carnivores [20, 36]. We included tiger sex as a covariate and used the Huggins closed capture model [37, 38] in R package *mra* [39] to estimate abundance. We tested for population closure using the program CloseTest [40]. We also used the two innate rates model (TIRM) in program CAPWIRE [41] with 10,000 iterative computations to estimate the population size and 95% confidence interval (CI).

To test the accuracy of the results produced by the capture-recapture method, we used an accumulation curve method to estimate population size by analyzing all of the genotyped scats collected in HC [42]. This construct is a function between the number of genotyped samples and the cumulative number of alien genotypes. The asymptotic value of the curve is the estimate of population size. In this study, we applied the Eggert model [43] in the program GIMLET to draw an asymptote of the function using 1,000 random permutations.

We also used Hawth’s analysis tools in ArcGIS 10.0 (available at http://www.spatialecology.com/htools/) to draw possible home ranges of some identified individuals using the minimum convex polygon. Geophysical and human infrastructure data in our map were obtained from the China fundamental geographic information dataset.

## Results

### Species Identification and Summary Statistics

We collected a total of 167 felid scat samples; 100 of them were collected using the routine sampling method in the three nature reserves from April 2013 to May 2015, and 67 of them were collected using the transect sampling method from December 2014 to February 2015 in HC (Table 1). Most scat samples were collected in HC and LYL within 20 km from the international border except for 3 scats that were collected in HNH (Fig 1). Among them, 92 scat samples were collected during the winter; the others were collected during other seasons. From all the samples, we identified 136 tiger and 3 leopard scats. Twenty samples failed in DNA amplification and eight samples were identified as dog. The success rate of species identification from routine sampling and line transect sampling were 72% and 95.5%, respectively.

**Table 1:** The total number of fecal samples, genotyping success, and individuals identified under two sampling methods in three nature reserves of Northeast China. The criterion for amplification was that at least nine of 10 loci were used to determine and select genotypes for individual identification.

Sampling method	Collected Samples	Tiger-positive samples	Selected genotypes	Identified individuals
Routine	100	72	34	9
Line transect	67	64	55	9
Total	167	136	89	11

### Genetic Variability and Individual Identification

The mean number of alleles (N_A_) of 10 microsatellite loci was 2.60, and the mean expected (H_E_) and observed (H_O_) heterozygosity were 0.42 and 0.49, respectively (Table 2). The mean PIC per locus was 0.35. Although only one locus (6HDZ089) was highly informative, most of the loci were reasonably informative. No significant deviation from Hardy-Weinberg equilibrium or linkage disequilibrium was detected among pairs of the 10 microsatellite loci. After multi-tube replication, 89 of 136 tiger-positive samples were successfully genotyped for more than nine loci, and the success rate of individual amplification was 65.4% (Table 1). The proportion successfully amplified from samples collected in the winter (77.2%) was noticeably higher than those collected in other seasons (49.1%).

**Table 2:** Characterization of 10 microsatellite loci for individual identification of Amur tigers. N_A_ = number of alleles per locus; H_O_ = observed heterozygosity; H_E_ = expected heterozygosity; PIC = Polymorphism Information Content; ADO = Allele dropout; FA = False allele.

Loci	N_A_	Size range (bp)	H_E_	H_O_	PIC	ADO	FA
FCA77	3	151–165	0.44	0.62	0.355	0.040	0.035
FCA94	2	209–211	0.38	0.24	0.310	0.056	0.004
D06	2	295–299	0.48	0.64	0.365	0.029	0.003
F42	2	205–233	0.50	0.78	0.374	0.040	0.007
FCA105	3	195–207	0.51	0.69	0.443	0.024	0.009
FCA441	3	149–157	0.52	0.68	0.437	0.067	0.032
FCA304	3	126–134	0.13	0.09	0.124	0.092	0.009
6HDZ089	3	211–223	0.57	0.55	0.512	0.069	0.017
FCA161	2	182–188	0.44	0.39	0.346	0.087	0.008
F124	3	208–220	0.21	0.16	0.189	0.094	0.002
Average	2.6	-	0.42	0.49	0.346	0.060	0.013

After comparing 89 multi-locus genotypes, 11 individual tigers (6 females and 5 males) were inferred to inhabit the study area (S1 Table). Of the identified individuals, 9 tigers each were identified from the routine sampling and the line transect sampling, and 7 of these tigers were detected using both sampling methods (S1 Table). Individual F1 was detected the most (29 times or 33% of the samples), whereas individuals F5 and M4 were recorded only once during the sampling period (S1 Table). Spatial-use patterns of resident breeding tigers or transient individuals, as well as the location of transects relative to the home ranges of individuals, are likely sources of heterogeneity in capture probability variation among individual tigers.

**Fig 2:**
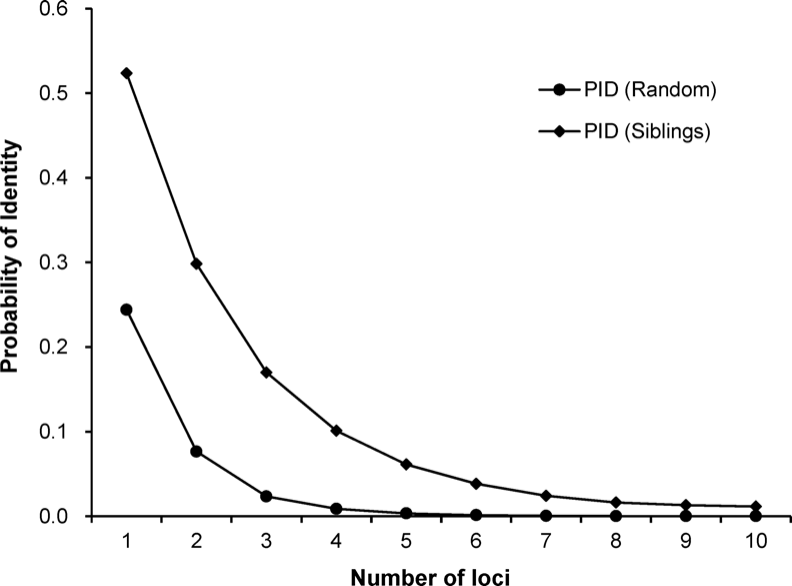
Variation in the probability of identity (PID) of unrelated individuals and siblings as a function of the 10 microsatellite loci used in this study. Loci were ordered from least to most informative.

GIMET analysis revealed that there was a high capability for distinguishing Amur tiger individuals when multiple loci were used. Despite the fact that several of the tigers may have been siblings, the error rate for incorrectly discriminating between them was as low as 1.15% when 10 loci were used (Fig 2). If the three most polymorphic loci (6HDZ089, FCA105 and FCA441) were omitted, the error rate for distinguishing siblings increased to 9.8%. The mean rate of ADO was 6.0%, whereas the mean rate of FA was 1.3% (Table 2). Therefore, the multi-tube approach can effectively reduce the error rate of genotyping and ensure reliability of genotypes. The mean samples per individual were 10.83 ± 10.61(SD) for females, 4.80 ± 4.66 (SD) for males. No significant difference was detected between the two sexes (Kruskal-Wallis test: χ^2^ = 1.22, df = 1, *p* = 0.27).

### Spatial Distribution

**Fig 3:**
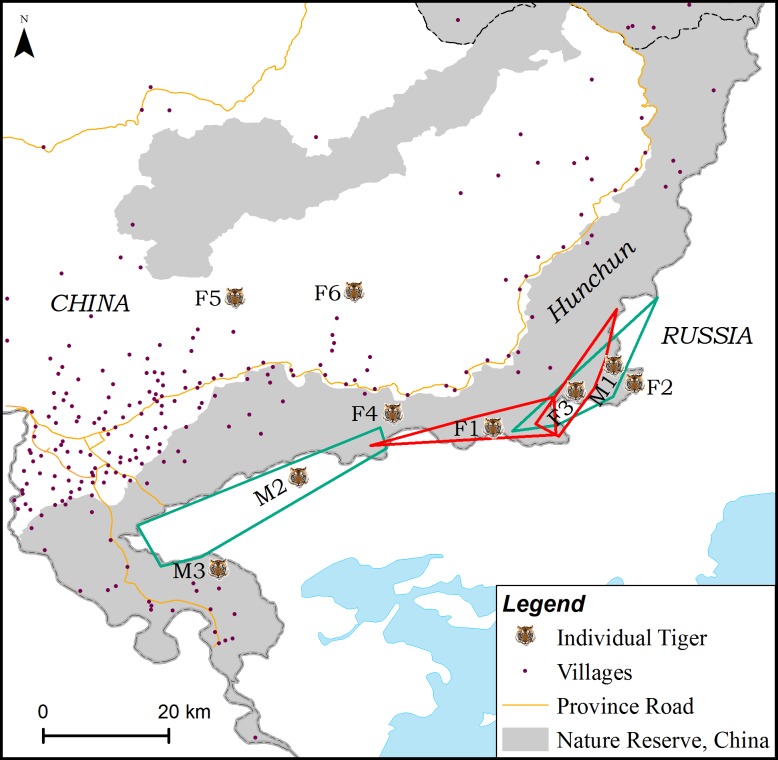
Location and home ranges of some tigers identified by genotyping scats collected from Hunchun Nature Reserve during 2013–2015. The green polygons are territories occupied by two male tigers, M1 and M2. The red polygons show the territories occupied by two female tigers, F1 and F3.

Most of the tigers (4 females and 3 males) were recorded in HC along the border with Russia during multiple surveys (Fig 3). Two female tigers F5 and F6 were identified in 2015 on the west side of the province road. One male was identified in 2014 in HNH, and another male was identified in 2013 and 2014 in LYL (not shown). The migration distance of two male tigers (M2 and M5) were far greater than that of any female tiger. The activity area of M1, a male tiger, covered the areas of female tigers F2 and F3 and that of another male, M2. The activity area of M1 may also cover the areas for both F1 and F4 (Fig 3).

### Population Size Estimation

**Fig 4:**
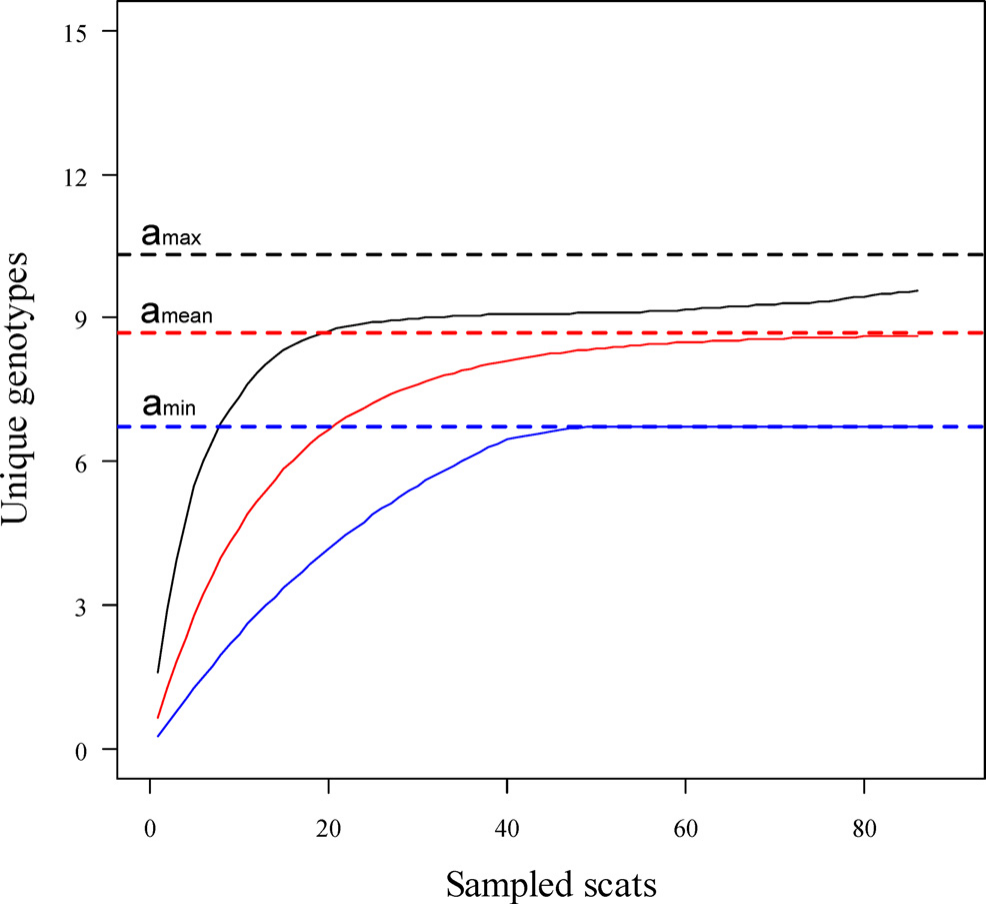
Population size estimation of Amur tigers in Hunchun Nature Reserve using the exponential curve of the Eggert model. Population size is represented by the asymptotic values of maximum (a_max_), mean (a_mean_), and minimum (a_min_).

The closure test supported the population closure assumption during the 54-day sampling period in the 2004–2005 winter (χ^2^ = 2.73, *p* = 0.91). Based on individual capture history (Table 3), population size was estimated to be 9 individuals (95% CI: 9–11) using the Huggins closed capture model in HC (Table 4). The TRIM model estimation was 9 (95% CI: 9–11). Using the Eggert model, we drew the fecal genotype rarefaction curve and estimated the population size of HC to be 9 ± 0.48 (SD) (Fig 4).

**Table 3:** Capture history and detection numbers for nine Amur tiger individuals sampled on nine occasions during the winter of 2014–2015. Every six days were considered a sampling occasion and every three occasions were combined as a time period to count the number of detections. F represents female tigers and M represents male tigers.

Individual	Capture history	Detection numbers			Total
		1	2	3	
F1	111111000	10	10	0	20
F2	011111010	3	7	1	11
F3	001101110	1	3	2	6
F4	001100000	1	1	0	2
F5	000000010	0	0	1	1
F6	000000011	0	0	2	2
M1	111010101	5	1	2	8
M2	110010100	2	1	1	4
M3	000000100	0	0	1	1
Total	-	22	23	10	55

**Table 4:** Amur tiger population estimate from a Huggins closed capture model and the two innate rates model (TIRM) during the winter of 2014–2015 in Hunchun Nature Reserve.

Model	Population size	SE	95% CI
Huggins	9	0.31	9–11
TIRM	9	-	9–11

## Discussion

### Individual Identification and Genetic Variability

Using non-invasively obtained genetic data, we could successfully identify 11 Amur tigers across the entire study area. The success rate of individual identification was higher (65.4%) than a similar study implemented in neighboring Russian reserves (58%) [23]. This increased success rate may also be due to the shorter time interval between sampling and DNA extraction in this study. In addition, the samples that we collected were all scats, but the noninvasive samples collected by Sugimoto et al. [23] included hair and saliva. The success rates of amplification of hair and saliva were lower than those for scats [35, 44].

Our results based on ten microsatellite loci suggest that Amur tigers show low levels of genetic diversity. The mean number of alleles and expected heterozygosity (N_A_ = 2.60, H_E_ = 0.42), which were consistent with results (N_A_ = 2.55, H_E_ = 0.44) obtained in the same area previously [25], were considerably lower than those of tigers in India (N_A_ = 3.33–12.43, H_E_ = 0.59–0.81) [24, 45, 46]. These values were slightly less than those of an adjacent population of southwest Primorye Krai (N_A_ = 3.20–3.56, H_E_ = 0.58–0.62) [23, 47] and the main Sikhot-Alin Mountain population in Russia (N_A_ = 2.39–3.33, H_E_ = 0.52–0.54) [7, 47]. Similarly, another study detected low genetic variation (wild population: H_E_ = 0.26; captive populations: H_E_ = 0.36) in a wide-scale analysis of Amur tigers [8]. These results are also in accord with earlier findings reporting the Amur tiger as having the lowest genetic variation of all extant tiger subspecies [1]. Historical and recent population bottlenecks may be the main reason for the low genetic diversity. By analyzing the genotypes of 95 individuals, Henry et al. [8] found genetic signatures of a historical bottleneck in the Amur tiger population, possibly due to the founder effect during postglacial colonization of its range some 10 000 years ago. Alasaad et al. [7] applied 11 loci to 15 individuals and detected a recent population bottleneck, which was attributed to the well-documented demographic collapse of the Amur tiger population in the 1940s.

Given that a barrier exists blocking movements of tigers between Hunchun-Southwest Primorye and the Sikhote-Alin Mountains, the small and isolated Hunchun-Southwest Primorye population may be at risk of continued genetic drift [8, 47]. The genetic structure of the small population further serves as a primary source for resettlement into the Changbaishan mountain forest complex of Northeast China, a process that is already underway [9, 10]. Hence, tiger conservation efforts should be targeted at a landscape-scale level, implementing an effective policy that includes further providing forested corridors that connect the entire subspecies and expanding current tiger reserves, especially in China, to minimize further genetic impoverishment, and ensure their long-term persistence.

### Spatial Distribution

Understanding the population dispersal and habitat connectivity is critical for wildlife conservation and management [48]. Historically, HNH once encompassed the entire distribution of the Amur tiger, but there were nearly no tiger tracks detected since the year 2000 [4, 49]. In 2014, our long-term camera trapping survey revealed that a young male tiger was photographed and traveled approximately 270 km from LYL to HNH [9]. This may be the first tiger to have migrated to this region since 2000. Meanwhile, in this study, one male tiger was identified from three scats collected from this region. We therefore speculate that this male tiger may be the same one identified by our genetic analysis. A prerequisite for animal dispersal is that the local habitat must be able to support a viable population and that interconnected corridors for animal dispersal exist [48, 50]. However, considering the small population size, the long distance, low forest connectivity and ubiquitous human activities between HNH and LYL, we inferred that the dispersal of the young male tiger was due to competition: this tiger had to move to locate a new territory because LYL and its surrounding areas had reached their maximum carrying capacity due to a low biomass of prey. At least 30 tiger individuals are now confined to approximately 4,000 km^2^ suitable habitat in Hunchun of China-southwestern Primorsky of Russia and have to share habitat with at least 80 leopards. Since Amur tigers also have large home ranges (401 ± 205 km^2^ and 778 ± 267 km^2^ for females and males, respectively) in this region, there is a resulting high overlap of home range size of adult males and the absence of territoriality [51]. These results suggest landscape conservation strategies must be a priority for both China and Russia to provide an opportunity for an increase in numbers of true residents on both sides of the border. We will continue to use DNA-based and camera-trapping techniques to examine tiger spatial dynamics in the future and to determine whether Amur tigers could settle and establish a new population, such as in HNH.

During the two year study period, almost all tigers were confined primarily to a narrow area along the border with Russia (Fig 3) in accordance with the observations of Wang et al. [10]. However, we were not able to conclude that the provincial road separating the HC and the forests of China interior are a barrier to tiger dispersal (Fig 1) because tigers were frequently found to cross the road and prey on domestic cattle [26]. Wang et al. [10] speculated that competition between domestic cattle and primary prey may be a major reason Amur tigers are straying out of HC in the west. Sugimoto et al. [23] concluded that the home ranges of Amur tigers in the southwest Primorye were also close to the border. Currently, however, to what extent tigers are using both sides of the border is not clear. Close collaboration between China and Russia in monitoring and conservation, along with wild prey recovery will be key in reestablishing the Amur tiger in Northeast China.

### Population Estimation

Our study provided a robust population estimate of Amur tigers in China using noninvasive genetic sampling techniques. The genetic capture-recapture method estimated a population size of 9–11 tiger individuals, which is similar to the result using the camera-trapping data in 2013 in HC where a population size of 11–13 Amur tigers was estimated [18]. The exponential curve of Eggert provided a similar estimate of population abundance, which confirmed the robustness of the results (Fig 4). We identified 9 individual tigers (6 females, 3 males) within a 3-month period in HC, whereas Chen et al. [17] reported only one female and two male tigers during the winter of 2009–2010. The comparison suggests that track surveys are of limited value in determining population size and sex of tigers. Our genetic data has revealed that a predominance of females now exists in HC, suggesting that HC is currently a critical site for the recovery of Amur tigers in China.

However, there are two reasons to be concerned about noninvasive genetic sampling methods. First, the abundance estimation may be biased because some individuals may be difficult to detect for behavioral reasons [19]. For instance, transient individual tigers rarely defecate on the residents’ territory to avoid detection, scat sampling may underestimate the size of the population. To overcome such biases, increased sampling intensity over a long time scale was used [19]. Second, there are several factors that affect the accuracy of the results of noninvasive genetic methods, such as sample size, the size of the sampling area, the quality of the DNA from the samples, the freshness of the scats collected in the field and the success rate of genotyping [20, 52]. For instance, the genotyping success rate of winter samples (77.2%) was much higher than that of samples collected during other seasons (49.1%). Freezing temperatures during the winter of Northeast China may greatly slow fecal DNA degradation. Therefore, noninvasive samples such as scat are in better shape when collected at low ambient temperature and when the DNA is extracted as soon as possible.

## Conclusion and Conservation Implications

This study provides a valuable independent population status assessment using genetic data from fecal DNA and demonstrates that noninvasive genetic sampling is an effective technique not only in obtaining estimates of population size but also for obtaining information on genetic diversity in species, such as the Amur tiger, for which limited data are available. This study has also established an important baseline for long-term monitoring programs for this charismatic species using the genetic capture-recapture framework in Northeast China. If repeated over multiple time periods, extensions of this framework allow vital rates to be estimated. These vital rates, in turn, drive changes in abundance.

Recovery of small populations of threatened species often relies on rigorous demographic estimates. This isolated small tiger population along the China-Russia border, which is at a viability threshold, is facing serious hurdles to dispersal and conflicts with local people [10, 26]. To facilitate tiger restoration in China, a possible solution may lie in mitigating human disturbances by minimizing the impact of cattle and humans on the forest landscape. Whatever interventions are implemented, monitoring changes in tiger abundance and tiger dispersal over time is essential. Our study is an illustration of a way in which population monitoring can be conducted for conserving Amur tigers. Finally, we recommend that sampling effort should be increased, that the geographic scale of sampling should be expanded, and that data from multiple surveys such as camera-trapping techniques or traditional snow tracking should be used together to improve population estimation [20, 21]. For example, Gopalaswamy et al. [53] combined scat data and camera-trapping data to obtain more precise tiger abundance and density estimates from both data sets.

## Supporting Information

S1 Table Frequencies of individual tiger scat for the routine opportunistic approach and the systematic transect approach, respectively, in Northeast China between April 2013 and May 2015. (DOCX)
