# Supplementary material for: Estimating the Population Size and Genetic Diversity of Amur Tigers in Northeast China
Source: PLoS One. 2016 Apr 21;11(4):e0154254. doi: 10.1371/journal.pone.0154254 (PMC4839643; doi:10.1371/journal.pone.0154254)
Supplement: S1 Table — (DOCX) [file pone.0154254.s001.docx]

**S1 Table. Frequencies of individual tiger scat for the routine opportunistic approach and the systematic transect approach, respectively, in Northeast China between April 2013 and May 2015.**

| Individual | Observation numbers | | Total |
| --- | --- | --- | --- |
|  | Routine sampling | Line transect sampling |  |
| F1 | 9 | 20 | 29 |
| F2 | 6 | 11 | 17 |
| F3 | 6 | 6 | 12 |
| F4 | 2 | 2 | 4 |
| F5 | 0 | 1 | 1 |
| F6 | 0 | 2 | 2 |
| M1 | 4 | 8 | 12 |
| M2 | 3 | 4 | 7 |
| M3 | 1 | 1 | 2 |
| M4 | 1 | 0 | 1 |
| M5 | 2 | 0 | 2 |
| Total | 34 | 55 | 89 |
